# Chloroplast ATP Synthase Modulation of the Thylakoid Proton Motive Force: Implications for Photosystem I and Photosystem II Photoprotection

**DOI:** 10.3389/fpls.2017.00719

**Published:** 2017-05-03

**Authors:** Atsuko Kanazawa, Elisabeth Ostendorf, Kaori Kohzuma, Donghee Hoh, Deserah D. Strand, Mio Sato-Cruz, Linda Savage, Jeffrey A. Cruz, Nicholas Fisher, John E. Froehlich, David M. Kramer

**Affiliations:** ^1^MSU-DOE Plant Research Lab, Michigan State University, East LansingMI, USA; ^2^Chemistry, Michigan State University, East LansingMI, USA; ^3^Cell and Molecular Biology, Michigan State University, East LansingMI, USA; ^4^Biochemistry and Molecular Biology, Michigan State University, East LansingMI, USA

**Keywords:** ATP synthase, proton motive force, *pmf*, photoprotection, PSI, PSII

## Abstract

In wild type plants, decreasing CO_2_ lowers the activity of the chloroplast ATP synthase, slowing proton efflux from the thylakoid lumen resulting in buildup of thylakoid proton motive force (*pmf*). The resulting acidification of the lumen regulates both light harvesting, via the q_E_ mechanism, and photosynthetic electron transfer through the cytochrome *b_6_f* complex. Here, we show that the *cfq* mutant of Arabidopsis, harboring single point mutation in its γ-subunit of the chloroplast ATP synthase, increases the specific activity of the ATP synthase and disables its down-regulation under low CO_2_. The increased thylakoid proton conductivity (g_H_^+^) in *cfq* results in decreased *pmf* and lumen acidification, preventing full activation of q_E_ and more rapid electron transfer through the *b_6_f* complex, particularly under low CO_2_ and fluctuating light. These conditions favor the accumulation of electrons on the acceptor side of PSI, and result in severe loss of PSI activity. Comparing the current results with previous work on the *pgr5* mutant suggests a general mechanism where increased PSI photodamage in both mutants is caused by loss of *pmf*, rather than inhibition of CEF *per se*. Overall, our results support a critical role for ATP synthase regulation in maintaining photosynthetic control of electron transfer to prevent photodamage.

## Introduction

Oxygenic photosynthesis is the most energetic biological process on earth and thus must be highly regulated to avoid self-destruction. This regulation is especially critical under conditions where light capture exceeds photosynthetic capacity, and thus leads to buildup of reactive intermediates that can produce deleterious side-reactions, e.g., when light intensity is increased or when metabolism is suppressed under environmental stresses. Plants ameliorate these effects through a series of feedback regulatory mechanisms that decrease the capture of light energy and modulate the transfer of electrons and protons. Some of the key regulatory mechanisms involve the electrochemical gradient of protons, or proton motive force (*pmf*) across the thylakoid membrane that is generated by light-driven electron transfer reactions through linear electron flow (LEF) and cyclic electron flow (CEF) (Strand and Kramer, 2014).

The *pmf* is energetically composed of two components, a proton concentration difference (ΔpH) from the translocation of protons by reduction and reoxidation of plastoquinone and the release of chemical protons from water oxidation—and an electric field (Δψ) from the vectorial transfer of electrons across the transthylakoid membrane (Avenson et al., 2004). Both ΔpH and Δψ drive the synthesis of ATP from ADP and inorganic phosphate (P_i_) at the chloroplast ATP synthase, but the ΔpH component has additional impact on regulating light capture and electron transfer reactions (Kramer et al., 2003). As ΔpH increases, the thylakoid lumen becomes more acidic, triggering the q_E_ response, which acts as a photoprotective mechanism by dissipating excess excitation energy from the light harvesting complexes to prevent over-excitation of photosystem II (PSII) (Müller et al., 2001). Lumen acidification also controls the oxidation of plastoquinol (PQH_2_) at the cytochrome *b_6_f* complex, limiting overall electron transfer preventing the buildup of electrons on photosystem I (PSI) electron acceptors (Takizawa et al., 2007).

Previous work has shown that the *pmf* is modulated by regulation of the chloroplast ATP synthase, providing critical regulatory connection between the light reactions and downstream metabolism (Kanazawa and Kramer, 2002; Kramer et al., 2004a). For example, when assimilation is limited by low CO_2_ availability, the activity of the ATP synthase is rapidly and reversibly decreased, slowing the efflux of protons from the thylakoid lumen (Kanazawa and Kramer, 2002; Avenson et al., 2005b; Kiirats et al., 2010), resulting in acidification of the lumen and initiating the down-regulation of the light reactions that involves activation of the photoprotective q_E_ response and slowing of electron transfer at the cytochrome *b_6_f* complex (Takizawa et al., 2007). Similar feedback regulation is observed under certain environmental stresses, e.g., drought stress (Kohzuma et al., 2009), by limitations in sink capacity at high CO_2_ (Takizawa et al., 2007) or when ATP synthase activity is decreased by mutations that affect its expression levels (Rott et al., 2011).

It has long been known that the chloroplast ATP synthase is regulated by modulation of a cysteine pair located in a regulatory loop in γ-subunit (Ort and Oxborough, 1992), which is modulated by light-induced electron flow from PSI through thioredoxin at high light and the chloroplast NAD(P)H: thioredoxin reductase C (NTRC) at low light (Carrillo et al., 2016). This regulatory mode is proposed to prevent wasteful ATP hydrolysis in the dark (Wu et al., 2007). Because reductive activation of ATP synthase occurs at very low light irradiance (Kramer and Crofts, 1989; Kramer et al., 1990) it has been suggested to be independent of metabolism-related regulation during active photosynthesis (Kanazawa and Kramer, 2002). Indeed, recent results from site-directed mutants of γ-subunit in Arabidopsis show that the redox- and metabolism-related regulation of ATP synthase acts via different mechanisms (Kohzuma et al., 2013) suggesting that metabolic intermediates, or post-translational modification of ATP synthase proteins (e.g., phosphorylation) mediate “metabolism-related” ATP synthase regulation.

It is also clear that the expression levels of the ATP synthase levels are regulated. For example, the ATP synthase content of the desert plant, wild watermelon, were shown to decrease substantially in response to drought stress, and this effect is likely important for activating lumen pH-dependent feedback regulation of photosynthesis under environmental stresses (Kohzuma et al., 2009). Schöttler and Tóth (2014) showed that ATP synthase content is, in most cases, regulated to match the capacity of the cytochrome *b_6_f* complex, and that this balance is critical to co-regulate electron and proton transfer reactions, though the mechanism of this regulation is not known. There is also evidence that a substantial fraction of the ATP synthase that is inactive, suggesting that secondary, or post-translational processes may further regulate its capacity by changing the fraction of active complexes (Rott et al., 2011), though this process has not yet been directly demonstrated.

Wu et al. (2007) isolated a mutant of Arabidopsis, *cfq* (‘*c*oupling *f*actor *q*uick recovery’), from an ethyl methanesulfonate (EMS)-modified library, with altered ATP synthase regulation. The *cfq* locus contains a missense mutation in atpC1 gene, resulting in a substitution of E244K in the γ_1_-subunit of ATP synthase. Arabidopsis possesses two γ-subunit homologes, γ_1_ and γ_2_, with the former being active in photosynthesis (Inohara et al., 1991; Dal Bosco et al., 2004) and the latter in non-photosynthetic tissues. For conciseness, we refer to the γ_1_-subunit as simply “γ-subunit.” The *cfq* mutation was reported (Wu et al., 2007) to shift the redox potential of the γ-subunit regulatory thiols, which should make it more sensitive to down-regulation in the dark. However, we report here that *cfq* ATP synthase also displays altered activity under steady-state photosynthetic conditions, likely by interfering with metabolism-related regulation, revealing a new role for regulation of the ATP synthase in preventing photodamage to both PSI and PSII.

## Materials and Methods

### Plant Strains and Growth Conditions

Wild type Arabidopsis (*Arabidopsis thaliana*), Columbia-0 (Col-0) and *cfq* were grown in a growth chamber in a 16:8 photoperiod with an average of 80 μmol photons⋅m^-2^⋅s^-1^ light at 23°C. This rather low light was maintained to prevent the accumulation of photodamage in the mutant lines. Plants between 17 and 23 days old were used for the experiments. Seeds for *cfq* were obtained from Prof. Donald Ort (Departments of Plant Biology and Crop Science, University of Illinois at Urbana-Champaign), and *npq4* were from Prof. Krishna Niyogi (Department of Plant and Microbial Biology, University of California at Berkeley). The M_3_ generation of the mutant *cfq* was back-crossed with Col-0 WT for three times, and the homozygous line was chosen from the F_3_ generation (Wu et al., 2007). A complementation line expressing wild type atpC1 behind a 35S promoter (35S::atpC1, or *comp*) was generated as described in Clough and Bent (1998) and Dal Bosco et al. (2004). This strategy for complementing point mutations was based on that presented in Wu et al. (2007) who observed ATP synthase phenotypes upon over-expressing mutant atpC1 variants in a wild type background, thus showing that the native γ-subunit can be outcompeted by other forms.

We used two complementary methods to confirm the expression of wild type atpC1 in *comp*, first by expression profiling with the insertion-confirmed lines, which showed the q_E_ value equivalent to the wild type in the Dynamic Environmental Photosynthetic Imaging (DEPI) system (Cruz et al., 2016), and second using a mass spectrometry approach. Briefly for this approach, chloroplasts from a single leaf from either Col-0, *cfq*, or complemented lines were isolated using Minute^TM^ Chloroplast Isolation Kit (Invent Biotechnologies, Inc., Eden, MN, USA), lysed and thylakoids were subsequently recovered by centrifugation. Chlorophyll content of thylakoids was determined by the method of Arnon (1949) to give a final concentration of 1 mg chlorophyll/ml for all samples. All thylakoids (5 μg chlorophyll total) samples were solubilized in Laemmeli buffer (Laemmli, 1970) and resolved by SDS-PAGE and stained with Coomassie Blue. Bands in the 37 kDa region (corresponding to the ATP-C protein) were excised and digested according to Shevchenko et al. (1996) and then subjected to mass spectrometry according to protocols established by the MSU Proteomics Facility. Eluted fragments were analyzed by Scaffold^TM^ 4.0 program. To monitor the rescue of *cfq*, we compared the recovery of certain fragments specific to either Col-0, *cfq* or rescued lines. A summary of this analysis is given in Supplementary Figure S7.

### *In Vivo* Spectroscopy Assays of Photosynthesis

Other chlorophyll fluorescence-derived photosynthetic parameters were obtained using the equations and methods described in Kramer et al. (2004b). The F_0_ point was measured in extensively (at least 30 min) dark adapted plants. The F_M_″ parameter, used to distinguish between q_E_ and q_I_, was measured after 3 min of dark adaptation following steady-state illumination. The dark interval relaxation kinetics (DIRK) of the electrochromic signal at 520 nm was for ECSt (*pmf*) and thylakoid proton conductivity (g_H_^+^) measurement (Avenson et al., 2005b; Baker et al., 2007). ECSt was calculated from the amplitude of the ECS signal during dark intervals of approximately 300 ms, and thylakoid proton conductivity (g_H_^+^) was estimated by fitting the ECS decay curve to a first-order exponential as previously described (Kanazawa and Kramer, 2002). The redox states of PSI were estimated from the difference in absorbance changes at 810 and 940 nm (Klughammer and Schreiber, 1994) with modifications described in the text and **Figure 4**.

### Protein Extraction and Western Blot Analyses

Two methods were used for protein quantification. For the result presented in Supplementary Figure S2, leaf samples were rapidly frozen in liquid nitrogen and ground in at 77 K with mortar and pestle, and suspended in an extraction buffer [100 mM Tricine-KOH, pH 7.5, 2 mM MgCl_2_, 10 mM NaCl, 1 mM ethylene-diamine-tetra-acetic acid (EDTA), 1 mM phenylmethylsulfonyl fluoride (PMSF), and β-mercaptoethanol]. The suspension was centrifuged at 13,000 rpm for 5 min, and the pellet was resuspended in a sample buffer [50 mM Tris-HCl, pH 6.8, 2% sodium dodecyl sulfate (SDS), 10 mM β-mercaptoethanol, 10% glycerol, and bromophenol blue]. Proteins were separated by 12% SDS-polyacrylamide gel electrophoresis (PAGE), and blotted onto polyvinylidene difluoride (PVDF) membranes (Invitrogen, USA). Blots were probed with polyclonal antibodies raised against β, γ, and 𝜀 subunits of ATP synthase, and cytochrome *f* (cyt *f*) of *b_6_f* complex. The western blots were exposed to films (Denville Scientific, Inc., Holliston, MA, USA) using ECL^+^ chemiluminescence kit (GE Healthcare, USA) with a peroxidase-conjugated anti-rabbit secondary antibody.

For the results presented in Supplementary Figure S5, total leaf tissue from *cfq* and Col-0 plants under control or fluctuating light were collected and homogenized using Minute-Chloroplast Isolation Kit (Invent Biotechnology, Inc. Plymouth, MN, USA) according to manufacturer protocol. Total leaf homogenates were quantitated for chlorophyll content and 5 μg chlorophyll/lane for each sample were analyzed by either SDS-PAGE or Western blot analysis. For western blots, proteins corresponding to 5 μg chlorophyll/lane were transferred onto PVDF membrane (Invitrogen) and probed with antibodies purchased from Agrisera (Vännäs, Sweden) according to manufacturer specifications [PsaB(AS10 695), CytF(AS10 695), PsaD(AS09 461), PsaF(AS06 104), PsbA(AS05 084A), and PC(AS06 141)] or produced in house (Tic110 and Toc75 using 1:5,000 dilutions). The detection method employed used a secondary anti-rabbit conjugated to alkaline phosphatase (KLP, Inc. Gaithersburg, MD, USA) at 1:5,000 dilution for 1 h in 5% DM/TBST. The blots were developed using a standard AP detection system with BCIP/NBT as substrates (Sigma-Aldrich, St. Louis, MO, USA). Bands were imaged and quantitated digitally using ImageJ (Rasband, 2008).

## Results and Discussion

### Growth Properties of *cfq*

Under typical low light used for Arabidopsis growth (to avoid photodamage), 16 h light: 8 h dark photoperiod with an average of 80 μmol photons⋅m^-2^⋅s^-1^ light at 23°C (for more details, see Materials and Methods), the *cfq* mutant grew with leaf area and thickness values that were indistinguishable from Col-0 (see Supplementary Figure S1). The chlorophyll content per leaf area was also indistinguishable (17.2 ± 0.5 μg Chl⋅cm^-2^ for Col-0, and 16.8 ± 0.7 μg Chl⋅cm^-2^ for *cfq*). Larger differences in growth phenotypes were observed when plants were grown for longer times under fluctuating illumination, as will be discussed in a forthcoming paper.

### Photosynthetic Properties of *cfq* Compared to Col-0

**FIGURE 1:**
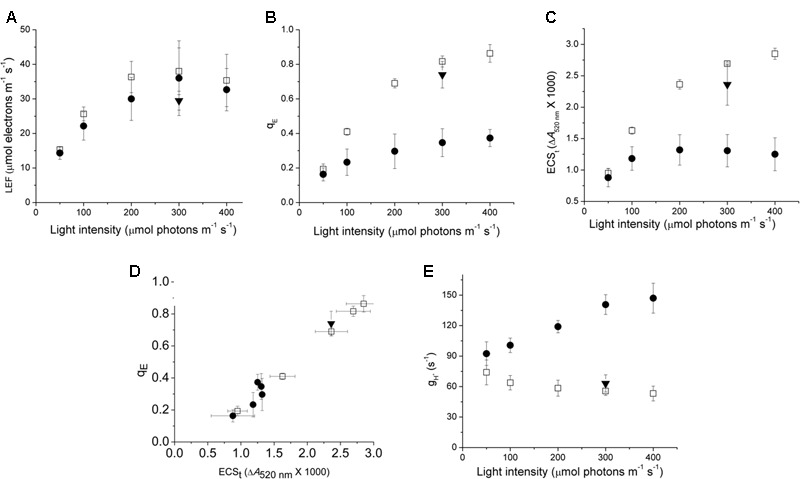
**Comparison of photosynthetic and photoprotective (q_E_) responses of Col-0 and *cfq* under ambient CO_2_.** Light dependence of linear electron flow (LEF, **A**), the q_E_ response **(B)**, the light-induced thylakoid *pmf* estimated by the ECSt parameter **(C)**; the dependence of q_E_ on the light-induced *pmf*, estimated by ECSt **(D)**, and the light intensity dependence of thylakoid membrane proton conductivity (*g_H_^+^*, **E**) reflecting ATP synthase activity. All experiments were conducted at 400 ppm CO_2_ levels, allowing at least 30 min exposure to each condition before measurements. Symbols indicate Col-0 (open squares), *cfq* (closed circles) and *comp* (35S::atpC1, closed triangles).

In order to understand the impact of variations in ATP synthase properties on the regulation of photosynthesis, we compared the photosynthetic response of Col-0, *cfq* and *cfq* 35S::atpC1 (*comp*) expressing wild type atpC1 in the *cfq* background. It is important to note that all experiments shown here were performed on plants that had been grown under non-stressed conditions. As will be discussed in detail in a forthcoming paper, exposing *cfq* plants to environmental stresses such as high light, drought, etc. results in long-term changes in the photosynthetic responses. In this work we focus on the short-term effects (from minutes to hours). The maximal PSII quantum efficiencies, measured by the fluorescence parameter F_V_/F_M_ taken just prior to our experiments were indistinguishable among *cfq*, Col-0, and *comp* (0.81 ± 0.2, 0.82 ± 0.2, and 0.80 ± 0.3), indicating that PSII was not damaged in the mutant prior to the experiments. **Figure 1** compares the responses of photosynthetic parameters of intact, attached leaves to a range of steady-state intensities of photosynthetically active radiation (PAR) under ambient (∼400 ppm) CO_2_. Photosynthesis was allowed to acclimate 30 min at each PAR to ensure steady-state conditions. In Col-0, LEF (**Figure 1A**) increased continuously with PAR, reaching half-saturation at approximately 100 μmol photons⋅m^-2^⋅s^-1^, and a light-saturated LEF of approximately 35 μmol electrons⋅m^-2^⋅s^-1^, consistent with previous results on Arabidopsis grown under these conditions (e.g., Livingston et al., 2010). There were no statistically significant differences in LEF among Col-0, *cfq* and *comp* at 300 μmol photons⋅m^-2^⋅s^-1^ (**Figure 1A**) but there were substantial differences in the q_E_ responses (**Figure 1B**). In Col-0 and *comp*, q_E_ increased continuously with PAR, reaching a value of about 0.6–0.8 at 300 μmol photons⋅m^-2^⋅s^-1^, comparable to previous results for plants grown under these conditions (Livingston et al., 2010).

In contrast, *cfq* showed q_E_ responses 2–3-fold smaller than in Col, reaching only 0.3 at 400 μmol photons⋅m^-2^⋅s^-1^, implying a loss of light-induced lumen acidification or q_E_-related antenna responses in *cfq*. The decrease in q_E_ response was largely reversed in *comp* and thus we attribute the major effects on photoprotection to the *cfq* mutation. It may seem surprising that changes in NPQ extent did not strongly affect LEF, but this effect is generally observed with moderate levels of NPQ seen in higher plants (e.g., Li et al., 2002; Brooks and Niyogi, 2011), because the NPQ dissipation of light energy competes with a very rapid capture of excitation energy by photochemistry; NPQ will, however, effectively decrease the quantum efficiency when photosynthesis is strongly limited by light intensity. In effect, the rate-limiting step for LEF is not at the level of light capture, but downstream at the level of the cytochrome *b_6_f* complex which in turn is controlled by lumen pH, so, that with moderate changes in NPQ, the PSII quantum efficiency (and thus LEF) is not strongly affected, but how the energy is lost from the photosynthetic apparatus is changed (Strand and Kramer, 2014). Inactivating q_E_ results in less NPQ, but in more PSII centers becoming closed by reduction of Q_A_, implies that primary function of NPQ is not to regulate electron transfer but to prevent the accumulation of reduced Q_A^-^_.

To distinguish between possible mechanisms for the loss of q_E_ in *cfq*, we probed the proton circuit of photosynthesis using *in vivo* kinetic spectroscopy. The DIRK of the electrochromic shift (ECS) can be used to monitor light-induced electron and proton transfer reactions that affect the thylakoid *pmf* (Avenson et al., 2005a; Baker et al., 2007). The amplitude of the ECS signal during the dark 0.5 s interval, termed ECSt, is related to the amplitude of light-driven *pmf*. The dependences of q_E_ on light-induced *pmf* (**Figure 1D**) for *cfq* and *comp* were all similar to that in Col-0, suggesting that the mutation did not substantially affect the partitioning of *pmf* into ΔpH and Δψ or the response of q_E_ to lumen acidification. If the fraction of *pmf* partitioning into ΔpH had increased, we would expect to see a higher extent of q_E_, which is responsive to lumen acidification, for a given total *pmf*, as we reported earlier (Avenson et al., 2005a; Baker et al., 2007). We thus conclude that the loss of q_E_ in *cfq* was most likely caused by decreased acidification of the lumen rather than altered antenna responses to lumen acidification.

The *cfq* mutant showed substantially lower amplitudes of light-induced *pmf* changes (as indicated by the ECSt parameter) (**Figure 1C**), implying an effect on proton efflux from the lumen rather than on light-driven proton transfer. This interpretation was confirmed by the data in **Figure 1E**, which shows that the loss of *pmf* in *cfq* can be explained by higher conductivity of the thylakoid to proton efflux (*g*_H_^+^), which in turn is attributable to the activity of the chloroplast ATP synthase (Kanazawa and Kramer, 2002; Avenson et al., 2005b). The fact that the decay of the ECS signal in leaves with inactive ATP synthase are similar in *cfq* and Col-0 (Wu et al., 2007) indicates that the signals we observe here are attributable to the ATP synthase activity and not to membrane leakage. The difference in *g*_H_^+^ was minimal at low light, but increased with light intensity, reaching about threefold higher values at 300–400 μmol photons⋅m^-2^⋅s^-1^, suggesting that the effect was related to the metabolic state of the chloroplast rather than to any basal increases in the rate of proton leakage. These results support the conclusion that the loss of q_E_ in *cfq* was caused by high ATP synthase activity that depletes the thylakoid *pmf*.

### The ATP Synthase in *cfq* Displays Altered CO_2_-Dependent Regulation that Affects Photosynthetic Control and Activation of Photoprotection

**FIGURE 2:**
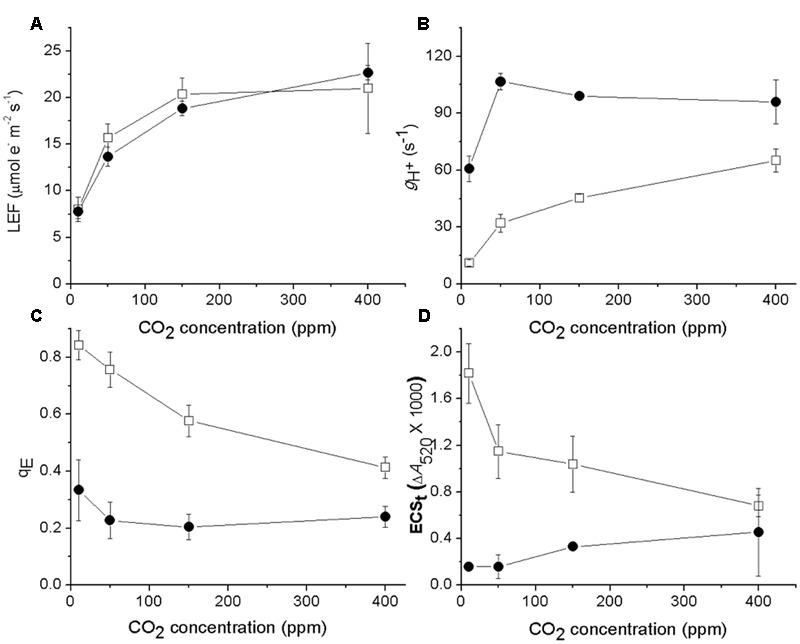
**Effects of CO_2_ levels on LEF (A)**, g_H_^+^ **(B)**, q_E_ **(C)**, and ECSt **(D)** with steady-state illumination at 100 μmol photons⋅m^-2^⋅s^-1^. Open squares and closed circles represent Col-0 and *cfq*, respectively.

We next tested if the responses of ATP synthase activity to CO_2_ levels were different in *cfq* by probing photosynthetic parameters during steady-state illumination (100 μmol photons⋅m^-2^⋅s^-1^) at ambient (400 ppm), low (50 ppm) and very low (less than 10 ppm) CO_2_ levels (**Figure 2**). Lowering CO_2_ led to decreases in LEF in both the wild type and mutant (**Figure 2A**), but the effect was somewhat larger in *cfq*, likely because of higher levels of photoinhibition (see below). In Col-0, decreasing CO_2_ from ambient to 10 ppm resulted in a progressive slowing of *g*_H_^+^, from 60 to about 12 s^-1^, likely reflecting decreased ATP synthase activity as previously described (Kanazawa and Kramer, 2002), with increased q_E_ responses (**Figure 2C**) resulting from higher *pmf* (**Figure 2D**). In contrast, *g*_H_^+^ in *cfq* was almost twofold higher at 400 ppm (96 s^-1^), and increased fractionally as CO_2_ was lowered to 50 ppm. Only at the lowest CO_2_ level did we observe a decrease in *g*_H_^+^ to about 60 s^-1^, close to that seen in Col-0 at 400 ppm. The q_E_ response of *cfq* roughly followed that of the *pmf*, and was largely independent of decreased in CO_2_ with a substantial increase at very low CO_2_. These results imply that the control or regulation of *g*_H_^+^, and thus likely the activity of the ATP synthase, is substantially altered in *cfq*.

### Evidence that the *cfq* ATP Synthase Has Specific High Activity

Supplementary Figure S2 summarizes results of western blot analysis of selected thylakoid membrane proteins in Col-0 and *cfq*, showing a loss of about 50% of ATP synthase proteins β, γ, and 𝜀 in *cfq* compared to Col-0. For comparison (load control), the levels of cytochrome *f* of the cytochrome *b_6_f* complex, and these were similar in Col-0 and *cfq*, as was consistent with the similar levels of photo-oxidizable cytochrome *f* measured in intact leaves in Col-0 and *cfq* (Supplementary Figure S3). Despite the fact that ATP synthase activity in *cfq* estimated by *g*_H_^+^ was equal to or higher than that in Col-0, the content of the ATP synthase proteins was about 50% lower than that of Col-0, suggesting that the existing ATP synthase had an effectively high specific activity. The reason for this effect is not known, but it suggests that the native ATP synthase operates below its potential maximal activity, perhaps as will be discussed below, as a regulatory mechanism. In this respect it is interesting that Rott et al. (2011) presented evidence that a substantial fraction of ATP synthase can be in inactive forms, giving a low effective specific activity, and one possibility is that the *cfq* mutation prevents the accumulation of this inactive form leaving to higher overall activity.

### Evidence that *cfq* Is Defective in Photosynthetic Control by the Cytochrome *b_6_f* Complex

**FIGURE 3:**
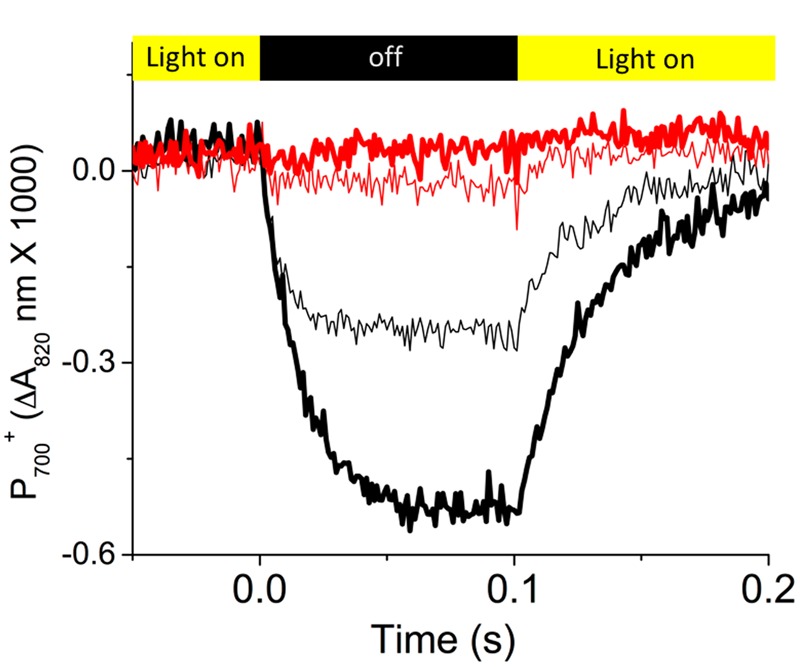
**Dark-interval relaxation kinetics reflecting the redox reactions of P_700_.** Prior to the measurements, attached leaves of Col-0 (black curves) and *cfq* (red curves) were illuminated for at least 20 min with 300 μmol photons⋅m^-2^⋅s^-1^ at ambient (400 ppm, thin curves) and low (50 ppm, thick curves) CO_2_. Kinetics of absorbance changes were measured using the IDEASpec device as described in the text. As shown in the colored bands at the top of the graph, the actinic illumination was switched off for 100 ms time intervals starting at time zero. Data is plotted as the fraction of P_700_ oxidized determined by the differences in absorbance changes at 810–940 nm, normalized to the maximum absorbance changes observed under far red illumination followed by a saturating flash.

**Figure 3** shows representative re-reduction kinetics of P_700_^+^ upon rapid light–dark transition from steady-state illumination (at 300 μmol photons⋅m^-2^⋅s^-1^) at ambient (about 400 ppm) and low (50 ppm) CO_2_ in Col-0 and *cfq*. In Col-0, at ambient CO_2_, P_700_ was re-reduced with a half time of about 5–7 ms, similar to previous results (Rott et al., 2011). Decreasing CO_2_ led to an increased extent of P_700_^+^ oxidation state and a slower rate of re-reduction (half time of about 25 ms) in the dark, likely reflecting the slowing of PQH_2_ oxidation with increased lumen acidity at low CO_2_ levels (Kanazawa and Kramer, 2002). In contrast, *cfq* showed very rapid P_700_^+^ re-reduction kinetics. At ambient CO_2_, P_700_ was only marginally oxidized and re-reduced rapidly with a half time of about 5 ms, though the precise time was difficult to quantify because of the small signal size. At 50 ppm CO_2_, no obvious P_700_^+^ re-reduction was observed during the dark interval, suggesting that in *cfq*, the rate of electron delivery to PSI was substantially more rapid than its oxidation by PSI photochemistry. In other words, the *cfq* mutant appears to have a constitutively rapid ATP synthase so that the normal buildup of ΔpH that initiates “photosynthetic control” of electron transfer at the cytochrome *b_6_f* complex is lost, so that re-reduction of P_700_^+^ is more rapid than its light-driven oxidation.

### The Redox State and Photoinhibition of PSI and PSII in Col-0 and *cfq* under Constant Light and Low CO_2_

**FIGURE 4:**
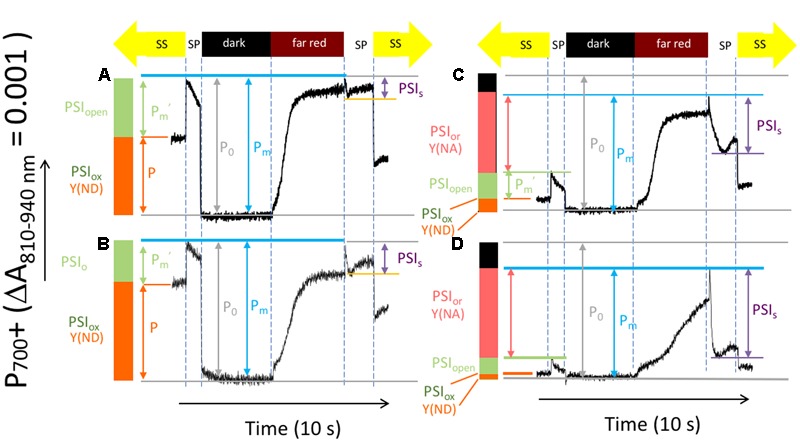
**Light induced P_700_^+^ absorbance changes reflecting the activity and redox state of PSI centers.** Attached leaves of Col-0 **(A,B)**, and *cfq* **(C,D)** under ambient **(A,C)** and 50 ppm CO_2_ **(B,D)**. Absorbance traces (at 810–940 nm) were taken during a series of illumination conditions, as illustrated in the timelines at the top of the graphs: (1) the steady-state actinic illumination for 30 min at 300 μmol photons⋅m^-2^⋅s^-1^ (labeled ‘SS’ at the top); (2) application of a ∼1 s intense light pulse (>10,000 μmol photons⋅m^-2^⋅s^-1^, labeled ‘SP’) to saturate PSI photochemistry; (3) a 5 s dark period to allow all P_700_^+^ to become reduced (‘dark’); (4) a 5 s period of illumination with far red light (>730 nm), which preferentially excites PSI over PSII (‘far red’), was given to slowly oxidize the PSI donors and acceptors; (5) a 2-s saturating light pulse to achieve near full oxidation of P_700_ (‘SP’ after ‘far red’); (6) return to steady-state illumination (‘SS’).

**Figure 4** shows representative kinetics traces reflecting light-induced P_700_/P_700_^+^ absorbance changes using a procedure modified from that introduced by Klughammer and Schreiber (1994) to test for changes in the redox state of PSI centers in Col-0 (**Figures 4A,B**) and *cfq* (**Figures 4C,D**) under ambient (**Figures 4A,C**) and 50 ppm CO_2_ (**Figures 4B,D**). Several parameters can be inferred from these experiments (Klughammer and Schreiber, 1994). Here, we extend or modify the established terminology (Klughammer and Schreiber, 2008) to avoid potentially confusing interpretations and to emphasize the special features relevant to the current data sets. For example, we observe large changes in the total photo-oxidizable P_700_ signal as the over the course of the experiment and between traces so that it is not possible to use this extent from individual traces as a reference point. We thus define our terms as follows, using parameters starting with P as in an indicator of P_700_^+^ absorbance signals and PSI referencing calculated PSI redox states:

(1) The total active PSI centers (PSI_act_) in the leaves under a certain condition is proportional to P_M_, the maximal absorption difference between dark and the second saturation pulse taken after application of far red light to oxidize electron carriers. It was assumed that all PSI centers in dark adapted material (before light treatments) were active, so the all parameters were normalized to the P_o_, P_M_ value measured in dark adapted leaves prior to illumination with a full complement of active PSI centers. Thus, for example, the fraction of active PSI, PSI_act_ is expressed as the ratio of P_M_/P_0_, where P_0_, is the P_M_ value measured in dark adapted leaves prior to illumination with a full complement of active PSI centers.
(2) The fraction of PSI centers with oxidized P_700_ under steady-state light, designated PSI_ox_ in **Figure 4** can be estimated from the parameter P, the absorption differences between the baseline (during steady-state illumination) and the dark interval (when all P_700_ was presumably reduced). This parameter is often termed Y(ND) for the quantum efficiency of thermal losses due to the presence of oxidized P_700_^+^. However, quantum efficiency parameters calculated from this type of measurement are potentially problematic because the derivations do not account for possible changes in PSI antenna efficiency or size. We thus prefer to use the less ambiguous terminologies that indicate PSI redox states.
(3) The fraction of PSI centers that were able to undergo charge separation under steady-state light, designated PSI_open_, can be estimated from absorption differences between the baseline (during steady-state illumination) and the first saturation pulse.
(4) PSI acceptor side limitations resulting from the buildup of electrons on PSI acceptors during the steady-state illumination, PSI over-reduced (PSI_or_), can be estimated from the difference in saturating pulse-induced absorbance changes taken in steady-state and after far red illumination. This parameter is sometimes termed Y(NA) for the quantum yield of exciton loss caused by acceptor side limitation.
(5) During the second saturation pulse the extent of P_700_^+^ can be seen to decrease, presumably as electrons accumulate on the acceptor side of PSI, preventing photochemistry and decreasing the extent of the 810 nm signal. We term the amplitude of this effect as PSI_s_.

Compared to Col-0, *cfq* showed strong suppression of both the steady-state P_700_^+^ oxidation state (PSI_ox_) and open PSI centers (PSI_o_) as well as an increase in the reduction state of the PSI electron acceptors, as indicated by an increase in PSI_or_ (**Figure 4**). These distinct responses likely reflect differences in the rate-limitations for electron transfer. In Col-0, electron flow was likely limited at the cytochrome *b_6_f* complex, which is typically controlled by acidification of the lumen, leading to net oxidation of P_700_^+^. In *cfq*, by contrast, electron flow appeared to be limited on the acceptor side of PSI, resulting in accumulation of electrons on stromal electron carriers and within PSI. Consistent with this view, lowering the CO_2_ resulted, in Col-0, in an increased in PSI_ox_, indicating a slowing of the *b_6_f* complex upon buildup of *pmf*. In contrast, in *cfq*, decreasing CO_2_ led to strongly *decreased* PSI_ox_ and *increased* PSI_or_, likely indicating a further reduction of the PSI electron acceptor pool. Also noteworthy was the large decrease in P_700_^+^ during the saturation pulse (PSI_s_), presumably reflecting the buildup of electrons in the stromal electron acceptor pool and subsequent inhibition of PSI photochemistry. Under our conditions, the extent of PSI_act_ was about 10 and 20% lower in *cfq* than in Col-0 under ambient and 50 ppm CO_2_, likely indicating a loss of active PSI centers in the mutant (see also below).

**FIGURE 5:**
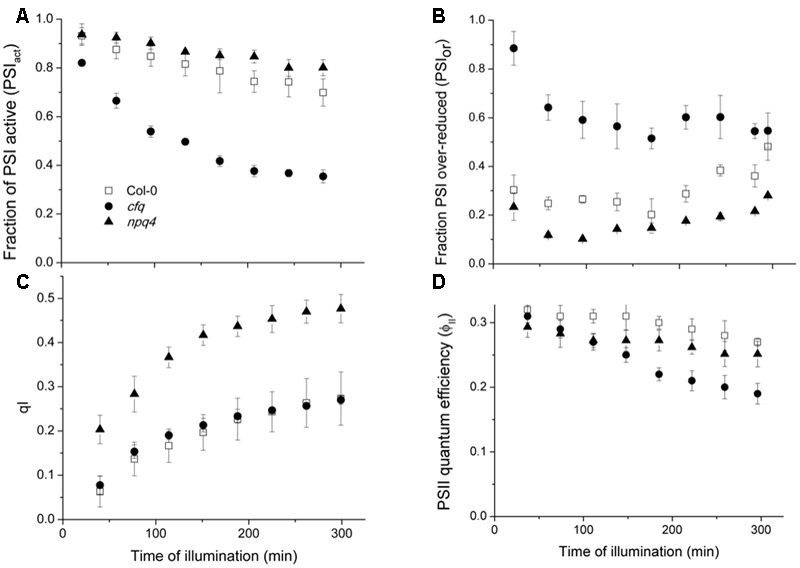
**Effects of illumination at low CO_2_ on PSI and PSII.** Attached leaves of Col-0 (open squares), *cfq* (closed circles) and *npq4* (closed triangles) were illuminated with 300 μmol photons⋅m^-2^⋅s^-1^ under 50 ppm CO_2_. Data are plotted as a function of time of illumination. **(A)** The fraction of active PSI (PSI_act_); **(B)** fraction of over-reduced PSI centers (PSI_or_); **(C)** PSII photoinhibition (q_I_); **(D)** PSII quantum efficiency (ϕ_II_).

To determine the effects of extended exposure to low CO_2_, we performed a time course for the PSI and PSII photosynthetic parameters, in Col-0, *cfq* and *npq4* (**Figure 5**). The *npq4* mutant was included to distinguish the effects of loss in *cfq* of q_E_ from that of decreased *pmf*, as discussed below. The extent and rate of PSI photoinhibition (**Figure 5A**), as reflected in the decrease in PSI_act_, were highest in *cfq*, which lost over 65% of photoactive PSI centers during the experiment; this effect was partially rescued in *comp*, see Supplementary Figure S4). By contrast, Col-0 lost less than 25% of active PSI. Interestingly, PSI in *npq4* appeared to be marginally *less* sensitive to these conditions, losing activity slightly more slowly. The trends in PSI inhibition were well-correlated with the extents of the PSI_or_ parameter (**Figure 5B**) as expected if over-reduction causes PSI photodamage as proposed by several groups (Terashima et al., 1994; Scheller and Haldrup, 2005; Suorsa et al., 2012; Allahverdiyeva et al., 2015). The highest extent of PSI_or_ was seen in *cfq*, at early time points accounting for over 80% of the total PSI centers, but decreasing to about 55% by the end of the experiment, possibly reflecting a decrease in delivery of electrons at later time points. In contrast, in Col-0, PSI_or_ remains at about 30% or lower throughout the experiment. The extent of PSII photoinhibition, estimated by the q_I_ parameter (**Figure 5C**), was but substantially higher in *npq4* compared to Col-0, as anticipated from previous studies (Brooks et al., 2014). In contrast, *cfq* showed very similar q_I_ responses to Col-0, suggesting that the stability of PSI, but not PSII was strongly compromised in *cfq* by illumination at low CO_2_. Interestingly, *npq4* showed the lowest extent of PSI_ox_, possibly reflecting decreased rates of delivery of electrons to PSI as a result of PSII photoinhibition, possibly reflecting the proposed role of PSII photoinhibition in protecting PSI (Tikkanen et al., 2014). The realized steady-state PSII quantum efficiency was most severely affected in *cfq*, implying that PSI photoinhibition had a stronger effect on overall photosynthesis.

### Effects of Fluctuating Light on *cfq*

**FIGURE 6:**
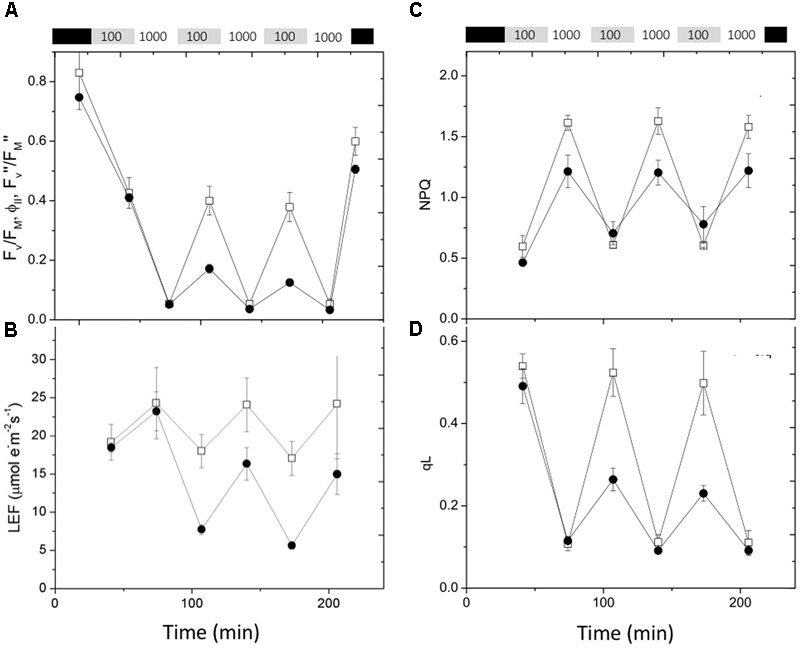
**Responses of chlorophyll fluorescence parameters to fluctuating illumination in Col-0 and *cfq*.** Plants were dark adapted for at 20 min and exposed to fluctuating light (30 min each of 100 and 1000 μmol photons⋅m^-2^⋅s^-1^) over a 4-h period. **(A)** Kinetics of quantum PSII efficiency, taken in dark adapted leaves (F_V_/F_M_), during steady-state illumination (ϕ_II_) and after 20 min of dark adaptation following light exposure (F_V_″/F_M_″); **(B)** LEF; **(C)** non-photochemical quenching (NPQ); and **(D)** Q_A_ redox state (q_L_). Symbols indicate Col-0 (open squares) and *cfq* (closed circles).

Rapid changes in actinic light intensity are known to severely impact photosynthetic processes and application of fluctuating light can reveal new photosynthesis-related phenotypes in mutants that would otherwise show only weak effects (Suorsa et al., 2012; Allahverdiyeva et al., 2015; Cruz et al., 2016). The data in **Figure 6** shows the effects on chlorophyll fluorescence parameters of exposure of previously dark-adapted leaves to fluctuating light alternating between 100 and 1,000 μmol photons⋅m^-2^⋅s^-1^ at 30 min intervals. The maximal quantum efficiency of PSII photochemistry in dark adapted leaves (F_V_/F_M_, **Figure 6A**) was slightly smaller in *cfq*, likely indicating the presence of a small extent of photoinhibition prior to the start of the experiment. During the first 30 min of 100 μmol photons⋅m^-2^⋅s^-1^ the steady-state PSII quantum efficiency (ϕ_II_) was similar in Col-0 and *cfq* (as seen in the experiments above), but much more strongly suppressed by exposure to high light (1,000 μmol photons⋅m^-2^⋅s^-1^). The loss of ϕ_II_ strongly decreased LEF, especially at higher light intensities (**Figure 6B**). During subsequent low light cycles, ϕ_II_ and LEF recovered in Col-0. In contrast, ϕ_II_ and LEF did not recover at low light in *cfq*, but rather showed a progressive decrease with each cycle of high and low light exposure. Despite this loss of activity, quantum efficiency in the dark (F_V_″/F_M_″) recovered to nearly the same extent Col-0 and *cfq* upon dark adaptation for 20 min, likely indicating that, although the mutant suffered some PSII photoinhibition, it likely did not account for most of the decreased electron transfer capacity during the low light conditions. Instead, it appears the loss of ϕ_II_ was related to decreased activity of downstream steps in photosynthetic electron transfer.

During the first low and high light cycle, NPQ was smaller in *cfq* than Col-0, consistent with the results above showing a loss of the q_E_ response in the mutant (**Figure 6C**). In subsequent illumination cycles, Col-0 showed a nearly constant pattern of increased NPQ at high light and return to lower values in the weak light. In contrast, *cfq* showed a gradual increase in NPQ with each cycle, consistent with a moderate increase in the rate of photoinhibition and loss of PSII activity, but not sufficient to account for the decrease in ϕ_II_ seen at low light. Instead, the strong decreases in the q_L_ parameter in the mutant (**Figure 6D**) indicate that PSII became limited by buildup of electrons on Q_A_, implying that electron transfer was blocked at a step following plastoquinone reduction, most likely at the acceptor side of PSI. The decrease in the q_L_ parameter increased throughout the remaining light cycles, suggesting that this blockage became progressively more restrictive with fluctuating light.

**FIGURE 7:**
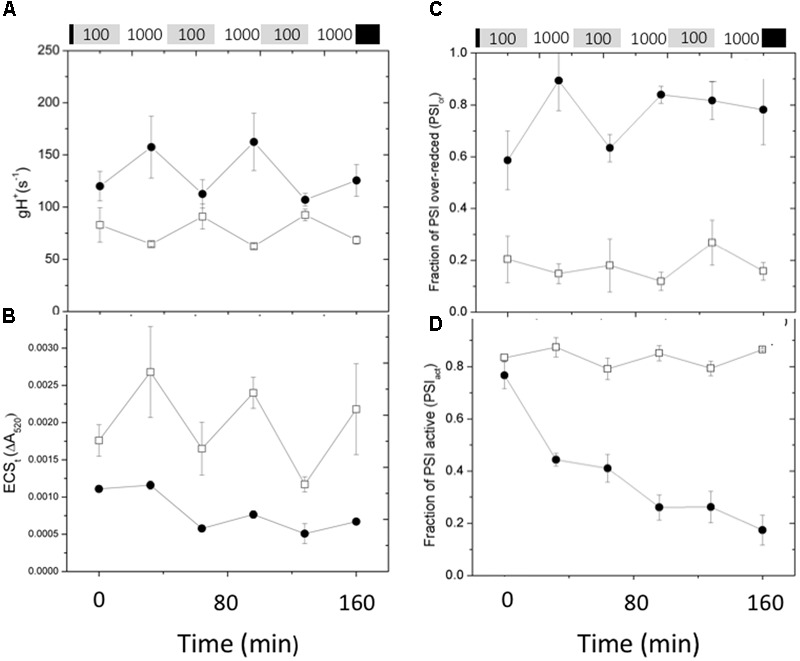
**Responses *pmf* and PSI to fluctuating illumination in Col-0 and *cfq*.** Plants were treated as in **Figure 6**. **(A)** Kinetics of the conductivity of the thylakoid membrane to protons (*g_H_^+^*); **(B)** ECSt, reflecting the light-induced thylakoid *pmf*. **(C)** Kinetics of the over-reduction state of PSI as estimated by the PSI_or_ parameter; **(D)** kinetics of deactivation of PSI centers, as estimated by the PSI_act_ parameter. Symbols indicate Col-0 (open squares) and *cfq* (closed circles).

**Figure 7** shows the effects of fluctuating light on ECS parameters, related to the generation and dissipation of the thylakoid *pmf*. The *g*_H_^+^ parameter (**Figure 7A**), was relatively constant for Col-0, increasing only slightly during the high intensity illumination, consistent with previous observations that the activity of the ATP synthase can be dependent on conditions such as CO_2_ levels, but relatively stable over this range of light intensity (Kanazawa and Kramer, 2002). During the first low light and high light exposures, *g*_H_^+^ was about 50% higher in *cfq* compared to Col-0, largely accounting for the small extent of the light-induced *pmf*, as estimated by the ECSt parameter (**Figure 7B**). Interestingly, the *g*_H_^+^ values increased in *cfq* during the higher light treatments, possible reflecting alterations in metabolic regulation. The amplitude of the light-induced *pmf* (ECSt) in *cfq* decreased with each successive light cycle, likely reflecting the combination of high *g*_H_^+^ and decreased light-induced electron and proton fluxes. These results suggest that the loss of ATP synthase control in *cfq* leads to a cascade of events under fluctuating light, starting with the light reactions and leading to secondary effects on downstream metabolic or regulatory processes.

**Figure 7** also shows the effects of fluctuating light on the redox state (**Figure 7C**) and PSI activity (**Figure 7D**). In Col-0, the extent of the PSI_or_ parameter was relatively constant at about 0.2 over the course of the experiment, indicating that even at high light electrons do not substantially accumulate on PSI acceptor side. In *cfq* by contrast, PSI was substantially over-reduced throughout the experiment, with PSI_or_ starting at 0.6 during the first low light treatment and increasing to about 0.9 during the first high light treatment, with only partial recovery in the following low light treatments. Overall, the results on fluctuating light are consistent with loss of *pmf* control of electron flow in *cfq*, leading to over-reduction of PSI and subsequent PSI photoinhibition.

Supplementary Figure S5 shows that the exposure to a 4-h fluctuating light regime (as in **Figure 6**) induced only small changes in the content of thylakoid proteins related to PSI (PsaD, PsaF, PsbA), PSII (PsbA), cytochrome *b_6_f* complex (cyt *f*) and plastocyanin (PC). To distinguish between PSI damage and loss of downstream electron acceptors, we infiltrated leaves with DCMU to block PSII and Methyl viologen (MV) as a rapid exogenous PSI electron acceptor and observed the extent and kinetics of P_700_ oxidation upon illumination (Supplementary Figure S6), comparing the effects of pre-exposure of leaves to 20 min of low light (100 μmol photons⋅m^-2^⋅s^-1^) or a single cycle of high light. The extent of photooxidizable P_700_ was lower in *cfq* than Col-0 and decreased strongly upon the high light treatment even though the major PSI proteins were unaffected, suggesting that while light treatment caused PSI damage, it did not result in loss of the PSI proteins that were assayed on the time scale of a few hours. This result suggests that the loss of PSI activity results from damage to the core electron transfer components, but not degradation of the PSI proteins themselves.

Interestingly, the photosynthetic phenotypes of *cfq* were strikingly similar to those of “proton gradient regulation 5” *pgr5* mutant. Munekage et al. (2002) ascribed the loss of *pmf* in *pgr5* to a defect in CEF around PSI and argued that this process was essential for maintaining photoprotection of PSII. The fact that both of these mutants show increased ATP synthase activity, leading to decreased lumen acidification leading to loss of q_E_ response, over-reduction of PSI (Avenson et al., 2005a; Suorsa et al., 2012) and severe PSI photodamage (Suorsa et al., 2012), suggests that the major function of PGR5 may be in adjusting the activity of the ATP synthase rather than regulating or catalyzing CEF.

## Conclusion

### The *cfq* Mutant Is Impaired in Down-Regulation of the ATP Synthase, with Critical Impact on the Responses of Photosynthesis to Changing CO_2_ Levels and Fluctuating Light

We describe a mutant, *cfq*, containing a single point mutation in the γ_1_-subunit of the chloroplast ATP synthase that affects the regulation and activity of the chloroplast ATP synthase. Previously, the *cfq* mutation was reported (Wu et al., 2007) to shift the redox potential of the γ-subunit regulatory thiols, which should make it *more* sensitive to oxidative down-regulation, i.e., one might expect that *cfq*-ATP synthase would have slower activity. Instead, we find that the activity of the *cfq*-ATP synthase as probed by *g*_H_^+^ was considerably faster than wild type-ATP synthase (**Figures 1E, 2B**) despite having a lower protein content of ATP synthase subunits (Supplementary Figure S2), suggesting that the specific activity of the ATP synthase is increased in *cfq*. The fact that *cfq*-ATP synthase activity responds differently to light (**Figure 1**), altered CO_2_ levels (**Figure 2**) and fluctuating light (**Figure 7**) suggests that the increased *g*_H_^+^ is caused by altered regulation or control of the ATP synthase. It is important to note that our results are not in contradiction with those of Wu et al. (2007) as the phenotypes we observed occur under higher or fluctuating lighting conditions.

Overall, these results are in broad agreement with lumen acidification being an essential component of the feedback regulatory system of light reactions, activating the photoprotective q_E_ response and governing the cytochrome *b_6_f* complex. The effects were minimal under permissive “laboratory-like” growth conditions but become particularly severe under low CO_2_ or fluctuating light, implying that ATP synthase regulatory control is an essential regulatory response of the light reactions to environmental or metabolic fluctuations. The loss of control of the thylakoid *pmf* during photosynthesis resulted in changes in the rate-limiting step in electron transfer, from the cytochrome *b_6_f* complex to the acceptor side of PSI, resulting in accumulation of electrons on PSI and subsequent PSI photodamage, possibly via the production of reactive oxygen species (Scheller and Haldrup, 2005; Rutherford et al., 2012; Huang et al., 2016; Takagi et al., 2016) that may result in destruction of the PSI iron sulfur complexes. Finally, the results suggest that engineering efforts to improve photosynthesis by increasing the rates of key rate-limiting steps in photosynthesis (e.g., the ATP synthase) can do more harm than good by short circuiting essential feedback regulatory systems.

## Author Contributions

Conceptualization: AK, EO, KK, DK; investigation: AK, EO, KK, DH, DS, MS-C, LS, JC, NF, JF, DK; original draft: AK, DK; writing – review and editing: AK, KK, DH, NF, JF, DK; funding acquisition: DK.

## Conflict of Interest Statement

The authors declare that the research was conducted in the absence of any commercial or financial relationships that could be construed as a potential conflict of interest.

## References

[B1] Allahverdiyeva Y. Suorsa M. Tikkanen M. Aro E. M. (2015). Photoprotection of photosystems in fluctuating light intensities. *J. Exp. Bot.* 66 2427–2436. 10.1093/jxb/eru463 25468932

[B2] Arnon D. I. (1949). Copper enzymes in isolated chloroplasts. Polyphenoloxidase in *Beta vulgaris*. *Plant Physiol.* 24 1–15. 10.1104/pp.24.1.1 16654194 PMC437905

[B3] Avenson T. Cruz J. A. Kramer D. M. (2004). Modulation of energy dependent quenching of excitons (qE) in antenna of higher plants. *Proc. Natl. Acad. Sci. U.S.A.* 101 5530–5535. 10.1073/pnas.0401269101 15064404 PMC397417

[B4] Avenson T. J. Cruz J. A. Kramer D. M. (2005a). Regulating the proton budget of higher plant photosynthesis. *Proc. Natl. Acad. Sci. U.S.A.* 102 9709–9713. 10.1073/pnas.0503952102 15972806 PMC1172270

[B5] Avenson T. J. Kanazawa A. Cruz J. A. Takizawa K. Ettinger W. E. Kramer D. M. (2005b). Integrating the proton circuit into photosynthesis: progress and challenges. *Plant Cell Environ.* 28 97–109. 10.1111/j.1365-3040.2005.01294.x

[B6] Baker N. Harbinson J. Kramer D. M. (2007). Determining the limitations and regulation of photosynthetic energy transduction in leaves. *Plant Cell Environ.* 30 1107–1125. 10.1111/j.1365-3040.2007.01680.x 17661750

[B7] Brooks M. D. Jansson S. Niyogi K. K. (2014). “PsbS-dependent non-photochemical quenching,” in *Non-Photochemical Quenching and Energy Dissipation in Plants, Algae and Cyanobacteria* Vol. 40 eds Demmig-Adams B. Garab G. Adams W. W. III Govindjee (Dordrecht: Springer). 10.1007/978-94-017-9032-1_13

[B8] Brooks M. D. Niyogi K. K. (2011). Use of a pulse-amplitude modulated chlorophyll fluorometer to study the efficiency of photosynthesis in Arabidopsis plants. *Methods Mol. Biol.* 775 299–310. 10.1007/978-1-61779-237-3_16 21863450

[B9] Carrillo L. R. Froehlich J. E. Cruz J. A. Savage L. Kramer D. M. (2016). The chloroplast NADPH thioredoxin reductase C (NTRC) is required for redox regulation of the chloroplast ATP synthase specifically under low irradiance. *Plant J.* 87 654–663. 10.1111/tpj.13226 27233821

[B10] Clough S. J. Bent A. F. (1998). Floral dip: a simplified method for *Agrobacterium*-mediated transformation of *Arabidopsis thaliana*. *Plant J.* 16 735–743. 10.1046/j.1365-313x.1998.00343.x 10069079

[B11] Cruz J. Savage L. Zegarac R. Kovac W. K. Hall C. C. Chen J. (2016). Dynamic environmental photosynthetic imaging (DEPI): continuous monitoring of genetic variations in photosynthetic response under dynamic growth environments. *Cell Syst.* 6 365–377. 10.1016/j.cels.2016.06.001 27336966

[B12] Dal Bosco C. Lezhneva L. Biehl A. Leister D. Strotmann H. Wanner G. (2004). Inactivation of the chloroplast ATP synthase gamma subunit results in high non-photochemical fluorescence quenching and altered nuclear gene expression in *Arabidopsis thaliana*. *J. Biol. Chem.* 279 1060–1069. 10.1074/jbc.M308435200 14576160

[B13] Hall C. Cruz J. Wood M. Zegarac R. DeMars D. Carpenter J. (2012). “Photosynthetic measurements with the idea spec: an integrated diode emitter array spectrophotometer /fluorometer,” in *Photosynthesis for Food, Fuel and Future* eds Kuang T. Lu C. Zhang L. (Beijing: Springer-Verlag) 184–189.

[B14] Huang W. Yang Y.-J. Hu H. Zhang S.-B. (2016). Moderate photoinhibition of photosystem II protects photosystem I from photodamage at chilling stress in tobacco leaves. *Front. Plant Sci.* 7:182 10.3389/fpls.2016.00182 PMC4761844 26941755

[B15] Inohara N. Iwamoto A. Moriyama Y. Shimomura S. Maeda M. Futai M. (1991). Two genes, atpC1 and atpC2, for the gamma subunit of *Arabidopsis thaliana* chloroplast ATP synthase. *J. Biol. Chem.* 266 7333–7338. 1826905

[B16] Kanazawa A. Kramer D. M. (2002). In vivo modulation of nonphotochemical exciton quenching (NPQ) by regulation of the chloroplast ATP synthase. *Proc. Natl. Acad. Sci. U.S.A.* 99 12789–12794. 10.1073/pnas.182427499 12192092 PMC130538

[B17] Kiirats O. Kramer D. M. Edwards G. E. (2010). Co-regulation of dark and light reactions in three biochemical subtypes of C4 species. *Photosynth. Res.* 105 89–99. 10.1007/s11120-010-9561-9 20549356

[B18] Klughammer C. Schreiber U. (1994). An improved method, using saturating light pulses, for the determination of photosystem I quantum yield via P700+ absorbance changes at 830 nm. *Planta* 192 261–268. 10.1007/s11120-010-9561-9

[B19] Klughammer C. Schreiber U. (2008). Saturation pulse method for assessment of energy conversion in PS I. *PAM Appl. Notes* 1 11–14. 10.1007/BF01089043

[B20] Kohzuma K. Cruz J. A. Akashi K. Hoshiyasu S. Munekage Y. Yokota A. (2009). The long-term responses of the photosynthetic proton circuit to drought. *Plant Cell Environ.* 32 209–219. 10.1111/j.1365-3040.2008.01912.x 19021886

[B21] Kohzuma K. Dal Bosco C. Meurer J. Kramer D. M. (2013). Light- and metabolism-related regulation of the chloroplast ATP synthase has distinct mechanisms and functions. *J. Biol. Chem.* 288 13156–13163. 10.1074/jbc.M113.453225 23486473 PMC3642356

[B22] Kramer D. M. Avenson T. J. Edwards G. E. (2004a). Dynamic flexibility in the light reactions of photosynthesis governed by both electron and proton transfer reactions. *Trends Plant Sci.* 9 349–357. 10.1074/jbc.M113.453225 15231280

[B23] Kramer D. M. Johnson G. Kiirats O. Edwards G. E. (2004b). New fluorescence parameters for the determination of QA redox state and excitation energy fluxes. *Photosynth. Res.* 79 209–218. 10.1023/B:PRES.0000015391.99477.0d 16228395

[B24] Kramer D. M. Crofts A. R. (1989). Activation of the chloroplast ATPase measured by the electrochromic change in leaves of intact plants. *Biochim. Biophys. Acta* 976 28–41. 10.1023/B:PRES.0000015391.99477.0d

[B25] Kramer D. M. Cruz J. A. Kanazawa A. (2003). Balancing the central roles of the thylakoid proton gradient. *Trends Plant Sci.* 8 27–32. 10.1016/S0005-2728(89)80186-0 12523997

[B26] Kramer D. M. Wise R. R. Frederick J. R. Alm D. M. Hesketh J. D. Ort D. R. (1990). Regulation of coupling factor in field-grown sunflower: a redox model relating coupling factor activity to the activities of other thioredoxin-dependent chloroplast enzymes. *Photosynth. Res.* 26 213–222. 10.1007/BF00033134 24420586

[B27] Laemmli U. K. (1970). Cleavage of structural proteins during the assembly of the head of bacteriophage T4. *Nature* 227 680–685. 10.1007/BF00033134 5432063

[B28] Li X.-P. Muller-Moule P. Gilmore A. M. Niyogi K. K. (2002). PsbS-dependent enhancement of feedback de-excitation protects photosystem II from photoinhibition. *Proc. Natl. Acad. Sci. U.S.A.* 99 15222–15227. 10.1073/pnas.232447699 12417767 PMC137571

[B29] Livingston A. K. Cruz J. A. Kohzuma K. Dhingra A. Kramer D. M. (2010). An *Arabidopsis* mutant with high cyclic electron flow around photosystem I (*hcef*) involving the NDH complex. *Plant Cell* 22 221–233. 10.1105/tpc.109.071084 20081115 PMC2828696

[B30] Müller P. Li X.-P. Niyogi K. K. (2001). Non-photochemical quenching. A response to excess light energy. *Plant Physiol.* 125 1558–1566. 10.1105/tpc.109.071084 11299337 PMC1539381

[B31] Munekage Y. Hojo M. Meurer J. Endo T. Tasaka M. Shikanai T. (2002). *PGR5* is involved in cyclic electron flow around photosystem I and is essential for photoprotection in *Arabidopsis*. *Cell* 110 361–371. 10.1104/pp.125.4.1558 12176323

[B32] Ort D. R. Oxborough K. (1992). In situ regulation of chloroplast coupling factor activity. *Plant Physiol.* 43 269–291. 10.1016/S0092-8674(02)00867-X

[B33] Rasband W. S. (2008). *ImageJ, U. S. National Institutes of Health*. Bethesda, MD: U. S. National Institutes of Health 10.1146/annurev.pp.43.060192.001413

[B34] Rott M. Martins N. F. Thiele W. Lein W. Bock R. Kramer D. M. (2011). ATP synthase repression in tobacco restricts photosynthetic electron transport, assimilation and plant growth by over-acidification of the thylakoid lumen. *Plant Cell* 23 304–321. 21278125 10.1105/tpc.110.079111 PMC3051256

[B35] Rutherford A. W. Osyczka A. Rappaport F. (2012). Back-reactions, short-circuits, leaks and other energy wasteful reactions in biological electron transfer: redox tuning to survive life in O-2. *FEBS Lett.* 586 603–616. 10.1016/J.Febslet.2011.12.039 22251618

[B36] Sacksteder C. A. Kanazawa A. Jacoby M. E. Kramer D. M. (2000). The proton to electron stoichiometry of steady-state photosynthesis in living plants: a proton-pumping Q-cycle is continuously engaged. *Proc. Natl. Acad. Sci. U.S.A.* 97 14283–14288. 10.1016/j.febslet.2011.12.039 11121034 PMC18910

[B37] Sacksteder C. A. Kramer D. M. (2000). Dark interval relaxation kinetics of absorbance changes as a quantitative probe of steady-state electron transfer. *Photosynth. Res.* 66 145–158. 10.1073/pnas.97.26.14283 16228416

[B38] Scheller H. V. Haldrup A. (2005). Photoinhibition of photosystem I. *Planta* 221 5–8. 10.1023/A:1010785912271 15782347

[B39] Schöttler M. A. Tóth S. Z. (2014). Photosynthetic complex stoichiometry dynamics in higher plants: environmental acclimation and photosynthetic flux control. *Front. Plant Sci.* 5:188 10.3389/fpls.2014.00188 PMC4026699 24860580

[B40] Shevchenko A. Wilm M. Vorm O. Mann M. (1996). Mass spectrometric sequencing of proteins silver-stained polyacrylamide gels. *Anal. Chem.* 68 850–858. 10.3389/fpls.2014.00188 8779443

[B41] Strand D. D. Kramer D. M. (2014). “Control of non-photochemical exciton quenching by the proton circuit of photosynthesis,” in *Non-Photochemical Quenching and Energy Dissipation in Plants, Algae and Cyanobacteria* eds Demmig-Adams B. Garab G. Adams W. W. III Govindjee (Dordrecht: Springer) 387–408. 10.1021/ac950914h

[B42] Suorsa M. Järvi S. Grieco M. Nurmi M. Pietrzykowska M. Rantala M. (2012). PROTON GRADIENT REGULATION5 is essential for proper acclimation of *Arabidopsis* photosystem i to naturally and artificially fluctuating light conditions. *Plant Cell* 24 2934–2948. 10.1105/Tpc.112.097162 22822205 PMC3426124

[B43] Takagi D. Takumi S. Hashiguchi M. Sejima T. Miyake C. (2016). Superoxide and singlet oxygen produced within the thylakoid membranes both cause photosystem I photoinhibition. *Plant Physiol.* 171 1626–1634. 10.1104/pp.16.00246 26936894 PMC4936555

[B44] Takizawa K. Kanazawa A. Cruz J. A. Kramer D. M. (2007). In vivo thylakoid proton motive force. Quantitative non-invasive probes show the relative lumen pH-induced regulatory responses of antenna and electron transfer. *Biochim. Biophys. Acta* 1767 1233–1244. 10.1104/pp.16.00246 17765199

[B45] Terashima I. Funayama S. Sonoike K. (1994). The site of photoinhibition in leaves of *Cucumis sativus* L. at low temperatures is photosystem I, not Photosystem II. *Planta* 193 300–306. 10.1016/j.bbabio.2007.07.006

[B46] Tikkanen M. Mekala N. R. Aro E.-M. (2014). Photosystem II photoinhibition-repair cycle protects photosystem I from irreversible damage. *Biochim. Biophys. Acta* 1837 210–215. 10.1016/j.bbabio.2013.10.001 24161359

[B47] Wu G. Ortiz-Flores G. Ortiz-Lopez A. Ort D. R. (2007). A point mutation in *atpC1* raises the redox potential of the *Arabidopsis* chloroplast ATP synthase gamma-subunit regulatory disulfide above the range of thioredoxin modulation. *J. Biol. Chem.* 282 36782–36789. 10.1016/j.bbabio.2013.10.001 17959606

