# Supplementary material for: Chloroplast ATP Synthase Modulation of the Thylakoid Proton Motive Force: Implications for Photosystem I and Photosystem II Photoprotection
Source: Front Plant Sci. 2017 May 3;8:719. doi: 10.3389/fpls.2017.00719 (PMC5413553; doi:10.3389/fpls.2017.00719)
Supplement: Supplementary file 1 [file Image_1.PDF]

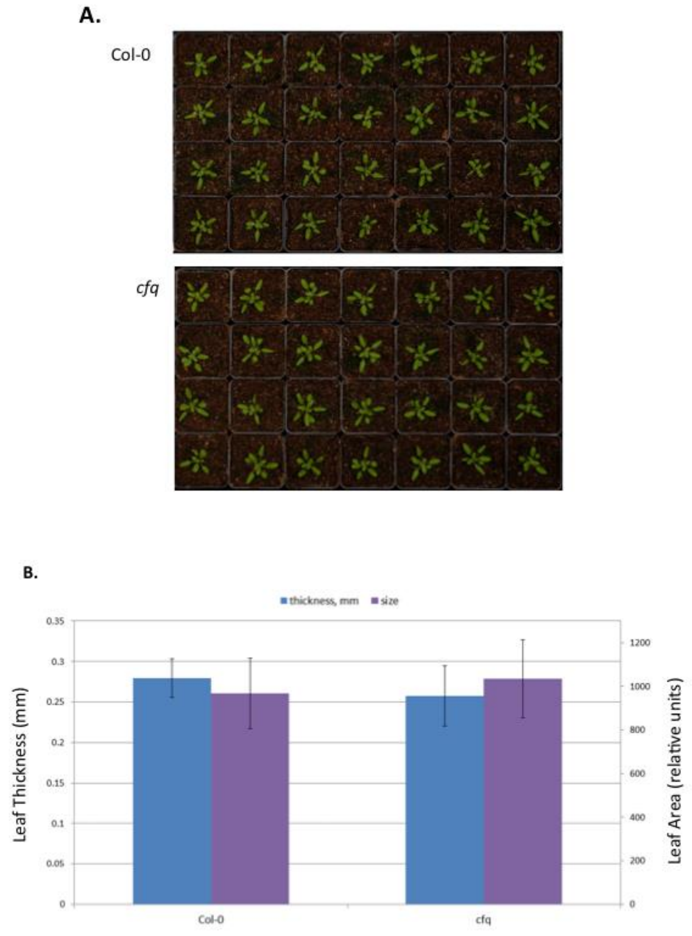

Supplemental Information Figure S1. Comparisons of leaf surface area (A) and leaf thickness (B) in Col-0 and *cfq* plants grown for 3-4 weeks under the conditions described in Materials and Methods. Leaf area measurements were based on pixel counts of fluorescence yield images measured during saturation pulses (F_M') using the ImageJ software package (Rasband, 2008).

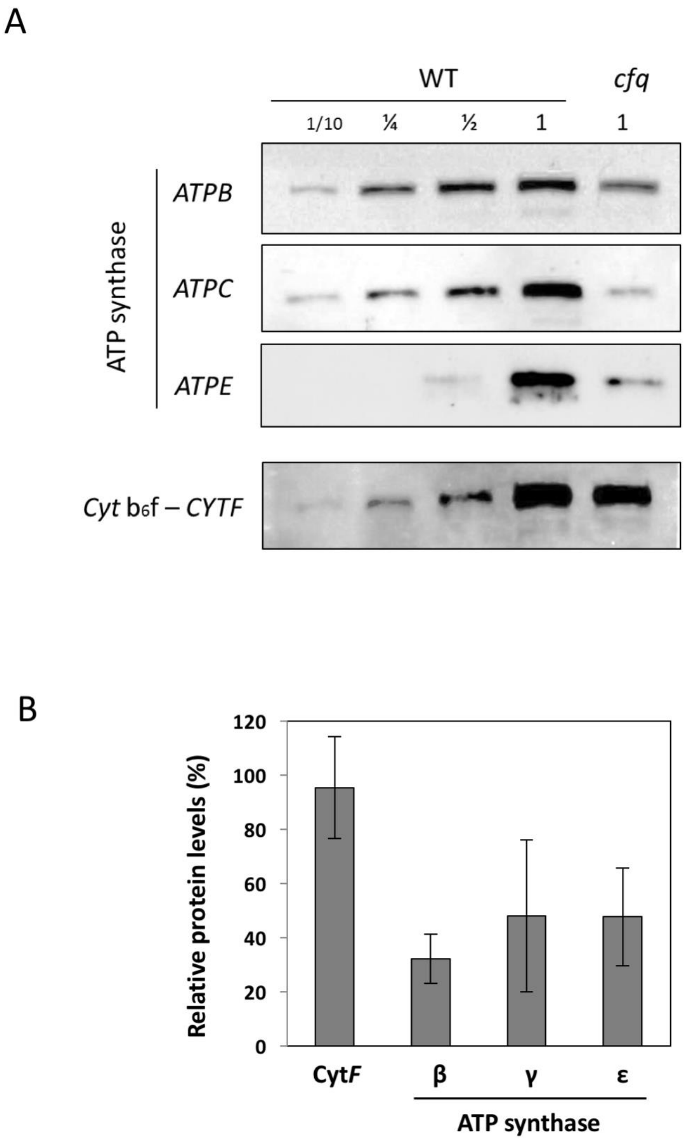

Supplemental Information Figure S2. Protein levels of chloroplast ATP synthase subunits and cytochrome *f* as in Col-0 (WT) and *cfq*. (A) Image of immunoblots of cytochrome *f*, and β (AtpB), γ (AtpC) and ε (AtpE) subunits of the chloroplast ATP synthase were performed on protein extracts from the leaves of wild type and *cfq.* (B) Chemiluminescent signals of each immunoblot in (A) were used to quantify changes in relative levels of the respective proteins in *cfq*, comparison with wild type. The signals in *cfq* proteins were calculated from loading dilutions of wild type, then the averaged values for the *cfq* detected by each antibody were set as 100% relative to the levels of proteins from wild type. Data are averages and standard deviation with *n* = 3.

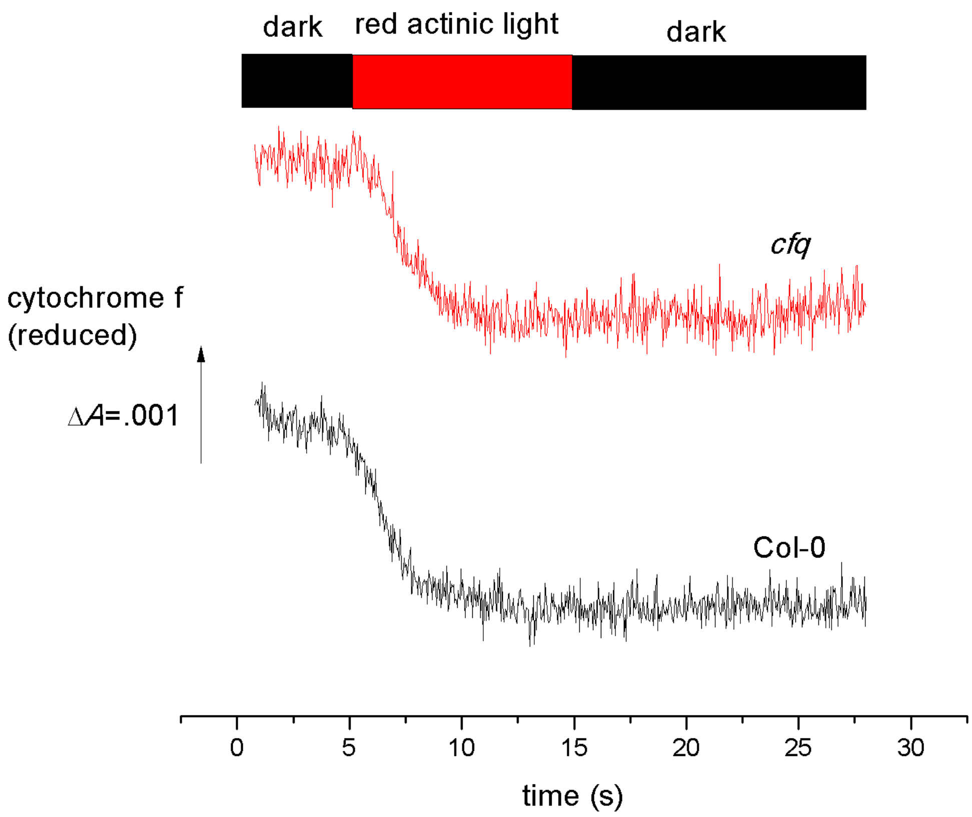

Supplemental Information Figure S3. The contents of photo-oxidizable cytochrome *f* were similar in *cfq* and Col-0. Detached fully expanded leaves were infiltrated with 10 µM DCMU (Sacksteder and Kramer, 2000) and dark adapted for at least 5 minutes followed by illumination with approximately 20 µmol photons$\cdot$m$^{-2}\cdot$s^{-1} red light. Shown are representative traces from n>3. Absorbance changes attributable to changes in the redox state of cytochrome *f* were recorded using the IDEASpec spectrophotometer (Hall et al., 2012) and deconvoluted as described in Sacksteder and Kramer (Sacksteder et al., 2000) except that results were expressed in Δ*A* rather than -Δ_I/I_0. Shown is a representative trace of n>3, showing similar extents of cyt *f* redox changes.

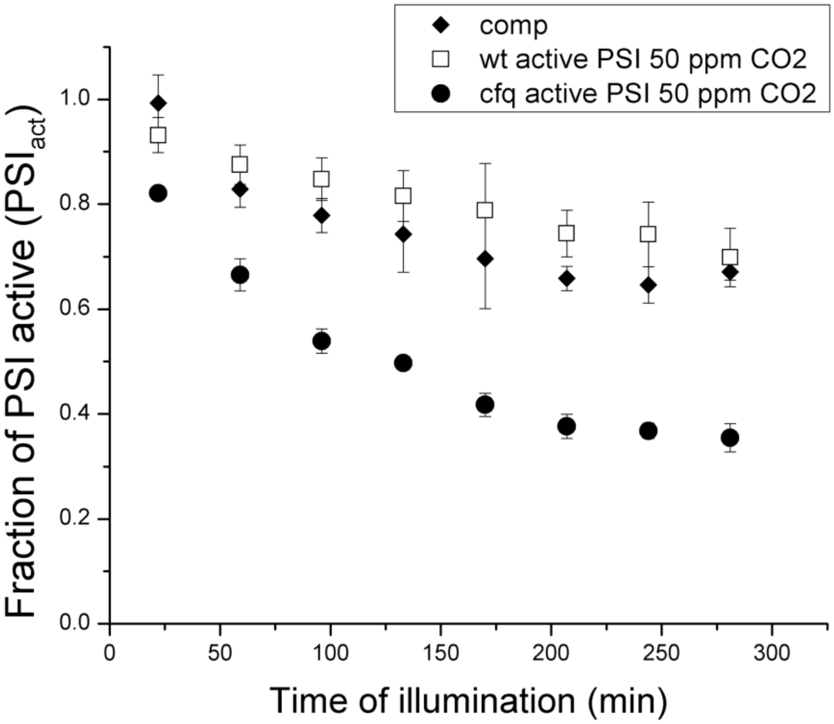

Supplemental Information Figure S4: Expressing atpC1 in *cfq* partially complements the sensitivity of PSI to photodamage. Experiments were performed on Col-0 WT (open squares) and *cfq* (closed circles) as in Figure 6, but additional traces were taken on *comp* (*cfq* 35S::atpC1, closed diamonds).

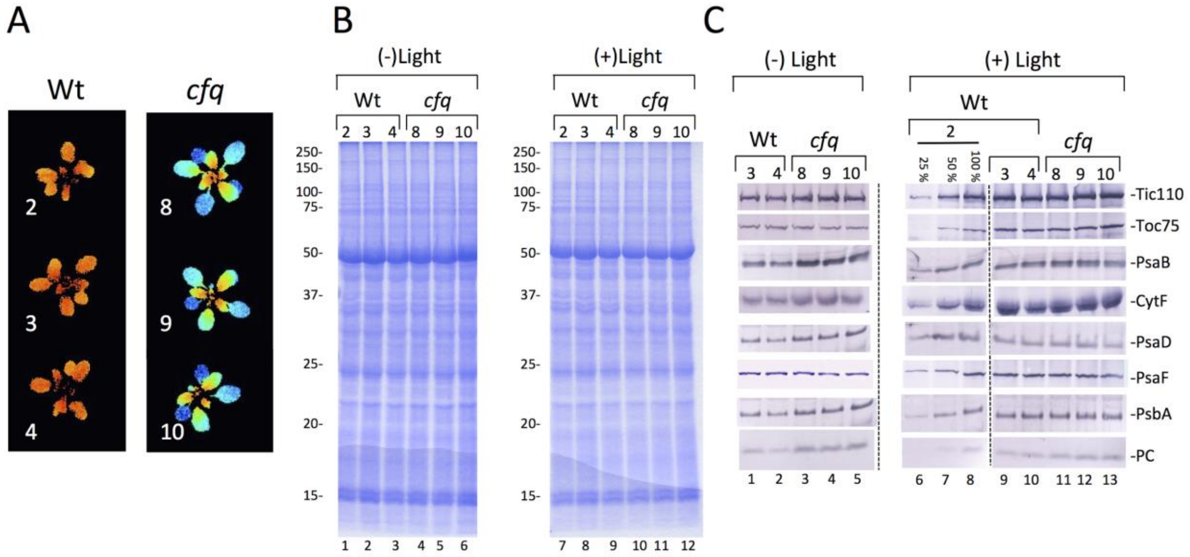

Supplemental Information Figure S5. Analysis of *cfq* and WT (Col-0) subjected to fluctuating high light conditions. (A) Dynamic Environmental Photosynthetic Imaging (DEPI) analysis (Cruz et al., 2016) of three independent lines of either Wt (Col-0) or *cfq* plants that have been subjected to fluctuating high light (1,000 µmol photons$\cdot$m$^{-2}\cdot$s^{-1}) treatment. (B) SDS-PAGE analysis of leaf tissue collected from plants in (A) first exposed to low light (100 µmol photons$\cdot$m$^{-2}\cdot$s^{-1}) [(-) Light] and then to fluctuating high light (1,000 µmol photons$\cdot$m$^{-2}\cdot$s^{-1}) [(+) Light]. 5 µg chlorophyll/lane was applied to a 12% SDS-PAGE gel and then Coomassie stained. (C) Western blot analyses of leaf tissue collected from plants in (A) were treated with antibodies against the indicated proteins as described in Materials and Methods.

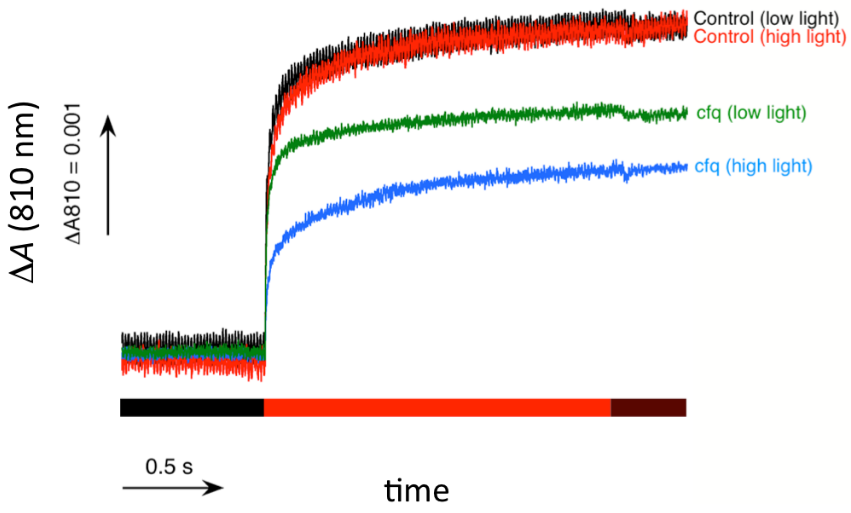

Supplemental Information Figure S6. Loss of PSI activity after exposure to fluctuating light measured by P_{700} oxidation extents. Leaves from the experiment were infiltrated with 50 µM DCMU (to block PSII) and 0.5 mM Methyl viologen (to prevent restriction of electron flow on the PSI acceptor side) and exposed to 500 µmol photons$\cdot$m$^{-2}\cdot$s^{-1} red actinic during and the 810 nm absorbance changes were measured.

A. Polyacrylamide gel from thylakoid preparations for isolation of ATPC1 proteins.

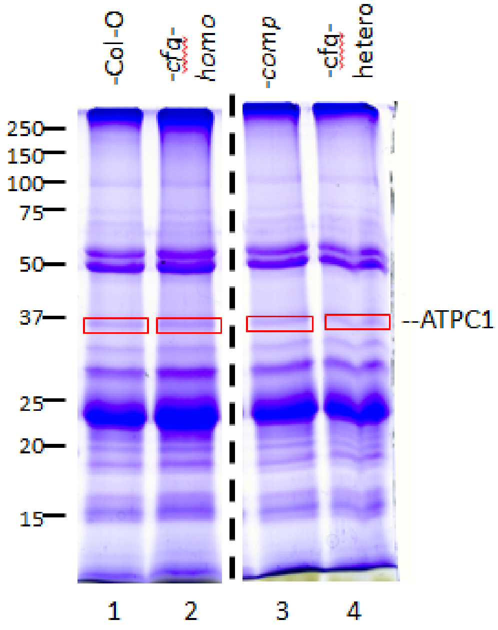

B. Sequences and expected distributions of mass-spec peptide fragments.

Fragment #	Amino Acid Sequence	WT	*cfq*
1	ALQESLASELAAR	Y	Y
2	TPTADFSPILQFEQDPVQILDALLPLYLNSQILR	Y	N
3	TPTADFSPILQFK	N	Y

C. Estimated ratios of mass-spec reads of peptide fragments.

Fragment reads (ratios)	WT	*cfq* homo	*cfq* hetero	*comp*
#2:#1	1.00	ND	0.55 (.02)	0.59 (.03)
#3:#1	ND	1.00	0.37 (.02)	0.38 (.01)
WT:*cfq*	1:0	0:1	1.5 (0.15):1	1.6 (0.12):1

Supplemental Information Figure S7. ATP synthase γ-subunit **expression in Col-0, homozygous and heterozygous *cfq* lines and *comp*** (35S::atpC1) **using a mass spectroscopic approach.** A) For each sample (See Lanes 1-4), Col-0 (WT), *cfq*, and *comp* (rescued *cfq*) lines

2-1 and 8-1, 5 μg total chlorophyll/lane from washed thylakoid preparations (see Arnon, 1949; Laemmli, 1970; Shevchenko et al., 1996) was loaded onto a 12.5% SDS-PAGE gel and then Coomassie stained. **Panel B. Sequences and expected distributions of mass-spec determined peptide fragments.** Because the mutation in *cfq* substitutes K for E at position 244, trypsin digestion should result in novel peptide fragments. Thus, to compare relative changes between various samples, we monitored the frequency of reads for certain fragments of γ-subunit that should be present in both WT and *cfq* (fragment 1), only in WT (fragment 2), and only in *cfq* (fragment 3). **Panel C. Estimated ratios of mass-spec reads of peptide fragments**. Bands at approximately 37 kDa (red box) corresponding to the ATPC1 protein were excised and subjected to mass spectroscopic analysis. To account for possible differences in fragmentation rates and other intensive factors, the reads of each fragment were normalized to those for fragment 1, expected to be represented in both WT and *cfq* isoforms, and then to these ratios in WT and *cfq* for fragments 2 and 3 respectively. The relative contributions for the WT and *cfq* forms were estimated from these normalized values (bottom row). Mass spectroscopy experiments for the mutant lines were performed n=3, with standard variation given in the parenthesis As expected, Col-0 and *cfq* samples showed reads exclusively for the WT- and *cfq*- specific forms of ATPC1. The heterozygous and *comp* lines showed reads from the Col-0 and *cfq* forms at ratios of about 1.5:1 and 1.6:1, respectively. These results in part confirm that expression of the WT form can outcompete the *cfq* form, partially complementing the mutant phenotype.
